# Picosecond Dynamics of Selective Ablation in a Ni/Al Multilayer Thin Film Induced by a Femtosecond Laser Pulse

**DOI:** 10.3390/nano16181168

**Published:** 2026-09-16

**Authors:** Sergey I. Ashitkov, Pavel S. Komarov, Evgenia V. Struleva, Mikhail A. Ovchinnikov, Dmitry S. Sitnikov

**Affiliations:** Joint Institute for High Temperatures, RAS, Izhorskaya St. 13 Bldg. 2, 125412 Moscow, Russia; komarov-p@yandex.ru (P.S.K.); struleva.evgenia@yandex.ru (E.V.S.); sitnik.ds@gmail.com (D.S.S.)

**Keywords:** multilayer thin-film, femtosecond laser pulse, interferometry, picosecond dynamics, spallation, phase explosion

## Abstract

In recent decades, high-precision processing with ultrashort laser pulses has been progressively applied in highly efficient surface modification technologies for new multilayer nanomaterials widely used in industry. In this work, the ultrafast dynamics of layer-by-layer ablation of a multilayer Ni/Al thin-film at the initial stage (0–120 ps) after irradiation with a single 60 fs Gaussian-shaped femtosecond laser pulse, together with the morphology of the modified surface, were investigated using single-shot spatiotemporal resolved interferometry. Partial removal of the 46 nm thick upper Ni layer took place in the spallation mode with an expansion velocity of several hundred meters per second. The lower spallation threshold observed relative to bulk Ni is attributed to interference of rarefaction waves, reflected from the layer boundaries. A jet-like strong ejection of material during the complete removal of the upper nickel layer in the phase explosion mode was accompanied by an explosive expansion of the underlying overheated molten aluminum. The experimental study was supported by calculations of the spatiotemporal behavior of temperature in thin-film layers. The obtained results may help in studying the ablation mechanism of multilayer thin-films, as well as in developing simulation methods and laser processing technologies.

## 1. Introduction

Laser processing of multilayer thin films using femtosecond laser pulses (FLPs) is relevant to a number of applications in microelectronics and photovoltaic devices [1,2,3]. Short irradiation times result in the formation of a small heat-affected zone, while modern laser and scanning systems allow material removal at speeds of several hundred meters per second. At the same time, the mechanisms governing selective femtosecond-laser processing of multilayer films with FLPs remain insufficiently understood. This is due to differences in the thermomechanical properties, thicknesses and sequences of the layers, thermal boundary resistance (TBR), and the interference of acoustic waves reflected from the interfaces. Previous studies have focused on the thresholds for selective ablation of layers and the morphology of the modified surface [4,5,6,7,8,9,10,11]. Depending on the coefficients of electron–phonon coupling and thermal conductivity, the absorbed FLP energy can be concentrated in either the upper or underlying layer of the structure [9]. Precise control of the pulse energy density is required to remove a thin top layer from an underlying one with a lower melting temperature [11]. The influence of layer thickness on the quality of the boundary associated with the localization of tensile stresses during laser selective processing was demonstrated in Ref. [12].

In recent years, interest has also been expressed in studying ultrafast ablation dynamics [12,13,14,15]. This is important for understanding the ablation mechanism and developing modeling methods for layer-by-layer removal [9,10,11,12,13,14], which can be used to design multilayer devices and improve the quality of their processing. In Refs. [12,13,14], a multi-pulse pump–probe femtosecond microscopy technique based on changes in the target’s reflectivity was employed to analyze thermal processes at the initial stage of interaction. However, the expansion of matter can only be observed after several hundred picoseconds using Newton’s rings imaging. In [15], multi-pulse imaging interferometry was applied to study ultrafast spallation dynamics of a thin gold film in the picosecond range.

By contrast, in the present study we applied ultrafast spatiotemporally resolved spectral interferometry using a chirped pulse along with Fourier processing of interference patterns [16,17]. This technique allows the continuous recording of not only changes in reflectivity, but also expansion of matter at the initial stage of laser–target interaction, from 0 to 200 ps, over a wide range of FLP energy densities in single-shot mode. Data obtained in this time range characterize the dynamics of ultrafast thermal and mechanical processes, the relaxation of absorbed energy, phase transitions, and spallation.

Increased interest in Ni/Al film structures is associated with exothermic reactions and the formation of intermetallic compounds [18], their processing [19] and use in memory and spintronic devices [20,21]. Previously, we studied a similar but not identical Ni/Al multilayer sample using *post-mortem* AFM and optical microscopy after single-shot irradiation with a tightly focused FLP (1028 nm, 270 fs), which provided only the ablation thresholds and final crater depths, without access to the expansion dynamics as such.

The main objective of this study was to investigate the ablation mechanism in the FLP-assisted separation of nanolayers to develop models and optimize the processing of thin films with lasers. This study presents new experimental data on the picosecond dynamics of selective layer removal in a multilayer Ni/Al film. Interferometric measurements were performed in single-shot mode over a wide range of laser fluence. A comparison of the results obtained through Fourier processing of the interference pattern’s spatiotemporal profiles of the amplitude and phase of the reflected probe pulse during surface expansion with the shape of the crater formed after laser irradiation indicates different ablation mechanisms for the first and second layers of the structure.

## 2. Materials and Methods

**Figure 1:**
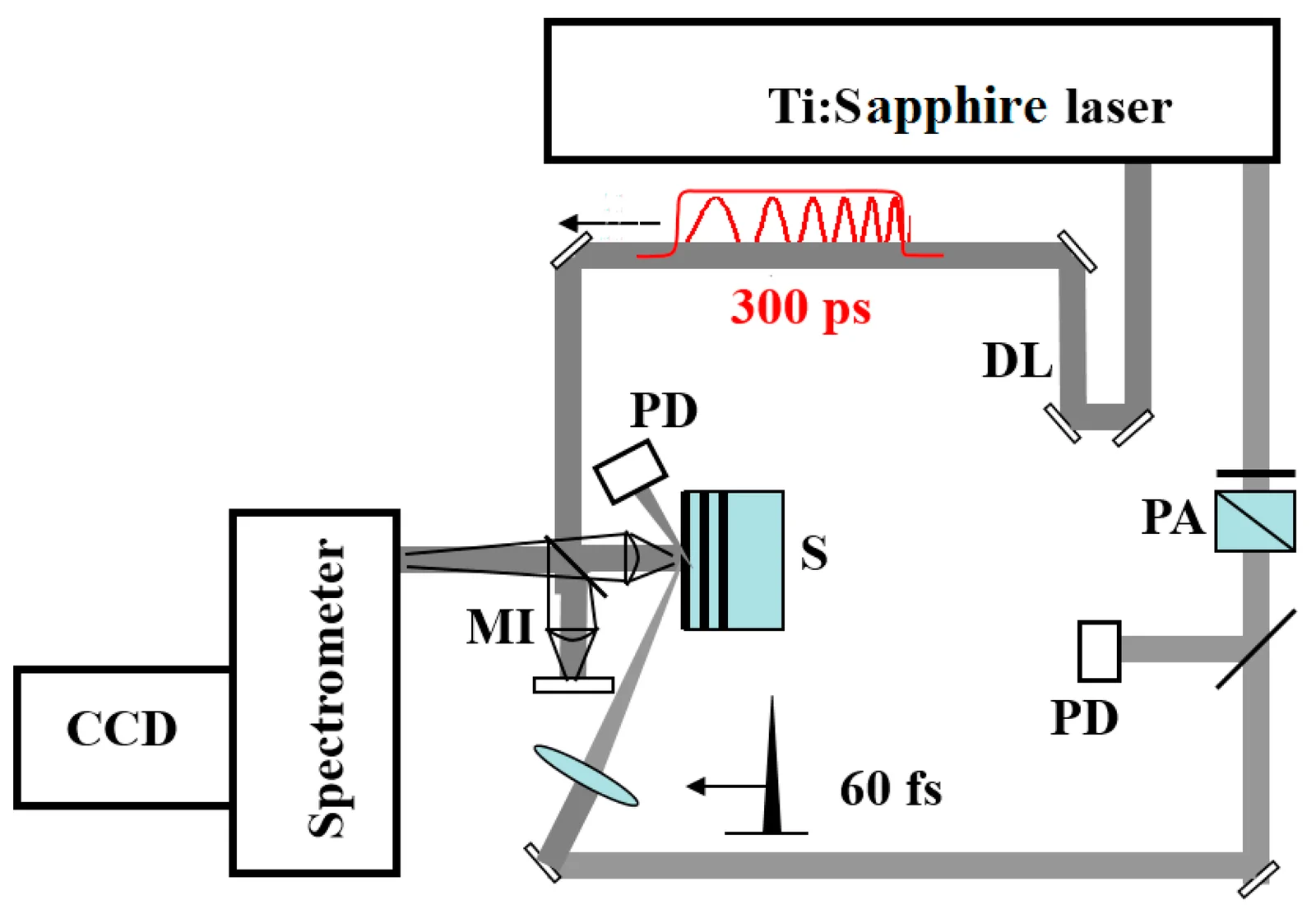
The experimental setup: DL—optical delay line; PA—polarizing attenuator; PD—photo detector; S—sample; MI—Michelson interferometer.

To study the picosecond dynamics of ablation, the pump–probe technique of spectral microinterferometry [16,17] was applied, which allows recording the continuous motion of the target surface and changes in its reflectivity under single-shot laser exposure with spatial and temporal resolution in the picosecond range. The scheme of the experimental setup is shown in Figure 1.

A Ti:Sapphire femtosecond laser system (Legend; Coherent Inc., Santa Clara, CA, USA) employing chirped pulse amplification served as the source of pump and probe pulses. An optical delay line ODL300/M (Thorlabs, Inc., Newton, NJ, USA) was used to match the moment of arrival of the pump and probe pulses at the target. A 60 fs *p*-polarized pump pulse at a wavelength of 800 nm was focused onto the target by a 20 cm focal length lens into an elliptical spot at a 60-degree incidence angle. The spatial distribution of the energy density in the focal spot *F*(*x*,*y*) was Gaussian with radii of *r*_0*x*_ = 60 ± 2 μm and *r*_0*y*_ = 25 ± 1 μm at the *e*^–1^ level. A widely used technique [22] was applied to measure the focal spot size, the maximum fluence $F_{0}=E/\pi r_{0x}r_{0y}$ in the center of the spot, and the ablation threshold values. The pulse energy was varied using a polarization attenuator and measured by a DET100A photo detector (Thorlabs, Inc., Newton, NJ, USA). Energy of the pump pulse reflected from the sample was detected by the 3Sigma energy meter (Coherent, Coherent Inc., Santa Clara, CA, USA). Given the specified uncertainties in *r*_0*x*_ and *r*_0*y*_, as well as the uncertainty in the pulse energy measurement Δ*E*/*E* = 2% (standard deviation, SD), the relative uncertainty in the determination of the laser fluence in the experiment was Δ*F*_0_/*F*_0_ ≈ 9% (SD).

The reflectivity of pump pulse from the multilayer Ni/Al film was measured shot-to-shot and found to be equal to *R* = 0.4, remaining constant within the investigated range of laser fluence from 0.1 to 1 J/cm^2^. This behavior is consistent with self-reflectivity measurements of nickel under 800 nm ultrashort-pulse excitation [23], which show that the reflectivity of Ni remains essentially unperturbed up to a fluence of about 1.0–1.6 J/cm^2^ for pulse durations of 50–100 fs, before decreasing above this threshold due to the contribution of fast-thermalized 4s-band electrons.

The probe was a frequency-modulated (chirped) pulse with a duration of 300 ps at λ = 800 nm. The diagnostic setup consisted of a Michelson interferometer combined with a grating spectrometer Acton 2300i (Acton Research Corporation, Acton, MA, USA) equipped with a 600-line/mm diffraction grating and a SensiCam QE CCD camera. A microlens with a numerical aperture of NA = 0.3 was used to transfer the image of the target surface to the camera.

A multilayered Ni/Al thin-film structure with a total thickness of 1.28 μm was deposited onto a 120 μm thick fused silica substrate for the investigations. The cross-sectional morphology of the film comprised a 400 nm thick Ni adhesion layer, above which 28 alternating Ni/Al bilayers with an individual layer thickness of 28 nm were stacked, and a top surface bilayer composed of Ni (46 nm) and Al (50 nm).

The thin-film structures were synthesized by direct-current (DC) magnetron sputtering without external substrate heating. The deposition chamber was evacuated to a base pressure of 10^−5^ Torr prior to the process. High-purity argon was then introduced as a working gas up to a pressure of 5 × 10^−2^ Torr. The Ni and Al magnetron targets were mounted on a rotating turret, which allowed their sequential alternation after the deposition of each individual monolayer without breaking the vacuum. To remove surface contaminants and native oxide layers from the target, a pre-sputtering procedure was performed for 500 s before the main deposition step.

For each measurement, three interferograms were recorded: *initial* (the undisturbed surface before laser exposure), *transient* (during exposure), and *final* (a few seconds after the exposure). Fourier processing of the interferograms was performed using an image normalization procedure [17,24]. The results of processing were the spatiotemporal distributions of the change in amplitude $A\left(t,y\right)/A_{0}$ and phase $∆\varphi (t,y)=\varphi (t,y)-\varphi_{0}$ of the reflected probe pulse. Here, *A*_0_ and $\varphi_{0}$ are the amplitude and phase values of the probe wave reflected from the surface of the undisturbed sample, respectively.

The amplitude and phase shift measurement errors were 2% and π/100, respectively. The surface displacement ∆z is related to the phase shift ∆φ by the following relationship:

(1) ∆z=∆φλ/4π.

The accuracy of displacement determination reached 2 nm, and the lateral spatial resolution was 5 μm. The temporal resolution, determined by the slit width and the spectrometer dispersion, was 2 ps.

No generative artificial intelligence has been used in this paper.

## 3. Results

**Figure 2:**
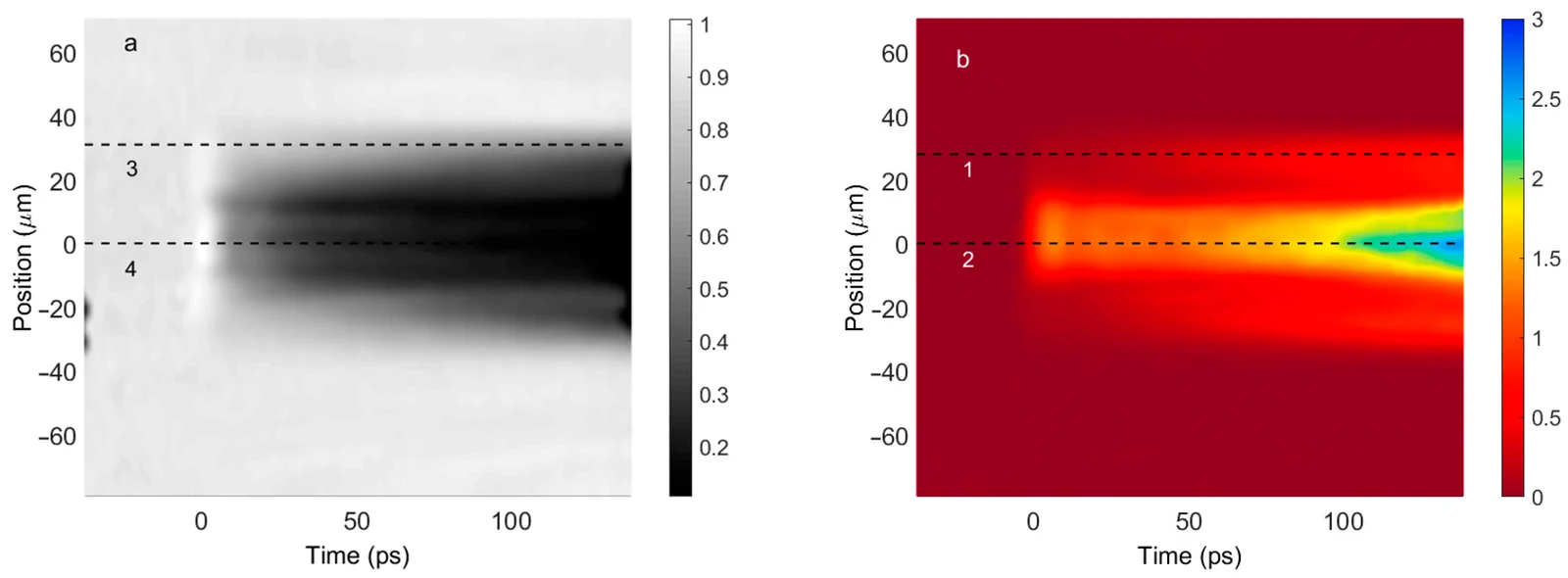
Spatiotemporal distribution of the amplitude (**a**) and the phase (**b**) changes in a probe pulse, reflected from the surface of the Ni/Al film at a pump fluence of *F*_0_ = 1 J/cm^2^. The color scale of phase shift in the right panel is given in radians. The dashed lines indicate the locations of the temporal cross-sections.

Figure 2 shows the spatiotemporal distributions of the amplitude $A_{t}\left(t,y\right)/A_{0}$ and phase $∆\varphi_{t}(t,y)$ changes in the probe pulse obtained from Fourier processing of the *transient* and *initial* interferograms.

**Figure 3:**
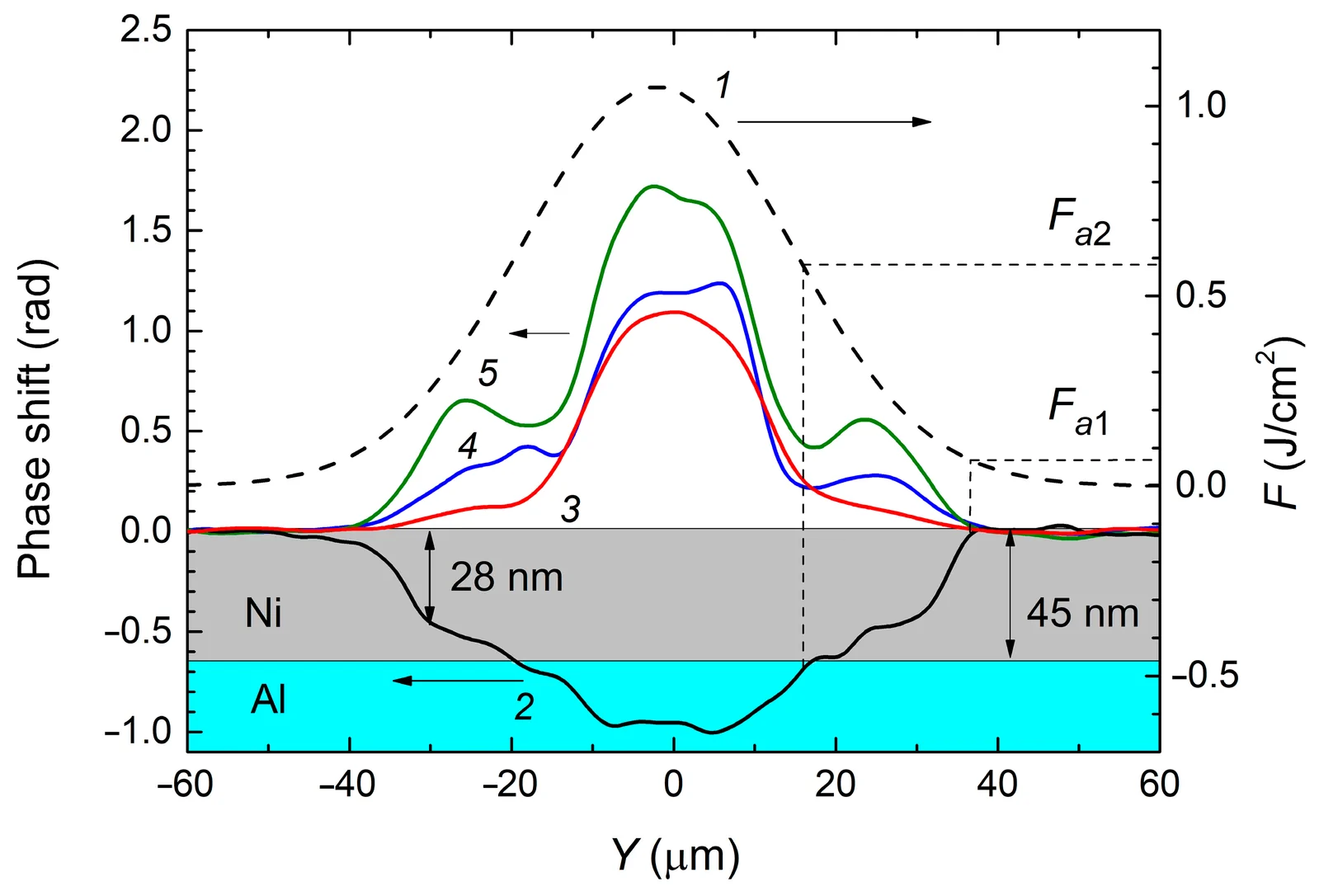
Gaussian energy density distribution in the focal spot (*1*); ablation crater profile (*2*); spatial profiles of phase changes at a delay of 2 ps (*3*), 43 ps (*4*), and 88 ps (*5*) for the Ni/Al film.

Figure 3 demonstrates the Gaussian spatial distribution of the pump energy density with *F*_0_ = 1 J/cm^2^ and a radius of *r*_0*y*_ ≈ 25 µm along the minor axis of an elliptical focal spot, as indicated by dashed line 1. The positive phase shift in Figure 3 corresponds to the expansion of the surface towards the detector, while negative values (curve *2*) describe the crater profile. Curve *2* shows the ablation crater profile formed on the film’s surface after exposure to an FLP. The crater profile was constructed after Fourier processing of the *initial* and *final* interferograms. The upper two layers Ni_1_ and Al_1_ of the multilayer structure are shown schematically in color.

After irradiation with a Gaussian beam with *F*_0_ = 1 J/cm^2^, complete or partial ablation of the layers occurred depending on the local fluence. The maximum depth at the crater center, according to relation (1) was *h*_max_ = 64 nm. The crater had a stepped shape. When the threshold value *F_a_*_1_ ≈ 0.07 J/cm^2^ was exceeded, a crater with a depth of 28 nm was formed on the surface of the film. As the energy density in the laser spot increased, so did the crater depth, eventually reaching the boundary between the layers at *F* ≈ 0.4 J/cm^2^. Ablation of the second layer Al_1_ occurred after exceeding the threshold value *F_a_*_2_ ≈ 0.6 J/cm^2^. The obtained value *F_a_*_1_ is consistent with the data in Ref. [10]. At the same time, it is several times smaller than the ablation threshold of a nickel bulk [25]. This difference is discussed below in Section 4.

Figure 3 also presents the spatial profiles of the transient phase shift $∆\varphi_{t}\left(y\right)$ at different times during the sample expansion process, extracted from the spatiotemporal distribution $∆\varphi_{t}(x,t)$ shown in Figure 2b. Initially, the profile $∆\varphi_{t}(y)$ has a shape similar to a bell (curve *3*). Over time, the profiles transform into a jet-like shape (curves *4* and *5*). The sharp increase in phase shift corresponds to the onset of removal of the underlying Al_1_ layer.

**Figure 4:**
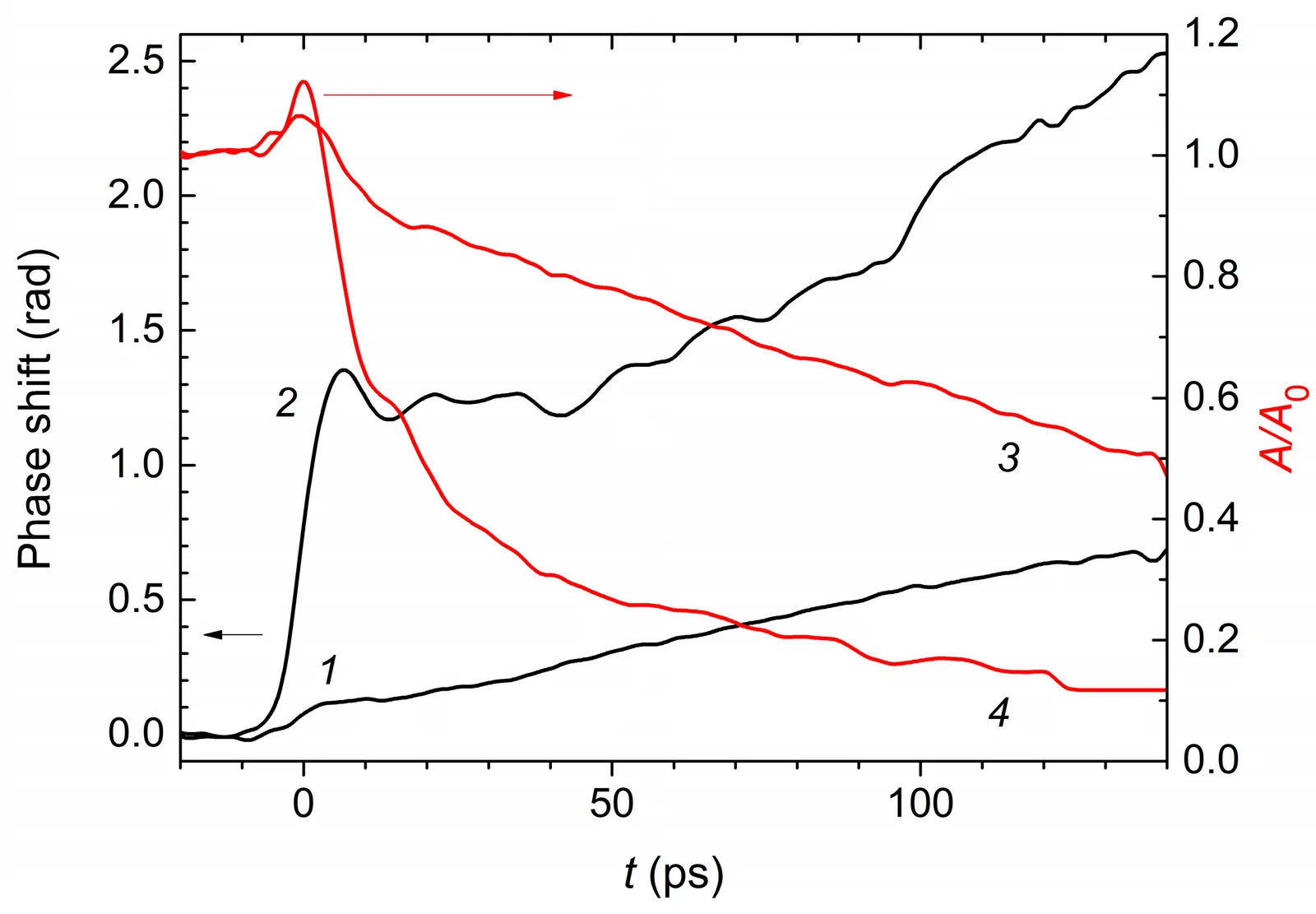
Temporal profiles of phase (*1*, *2*) and amplitude (*3*, *4*) changes at different fluence: *1*, *3*—0.2 J/cm^2^; *2*, *4*—1 J/cm^2^. Line numbers correspond to cross-sections in Figure 2.

Figure 4 presents the temporal dependences of the phase shifts (curves *1* and *2*) and amplitude (lines *3* and *4*) changes in the probe pulse, describing the dynamics of layers expansion. The cross-section locations are shown in Figure 2 by dashed lines under the corresponding numbers.

The profiles are plotted at different distances from the center of the impact area and correspond to different FLP fluence in the focal spot. The chosen zero point (*t* = 0) coincides with the reflection peak (curve *4*), which corresponds to the maximum electron temperature *T_e_* achieved during FLP exposure. Such reflection behavior is associated with interband transitions in nickel in the near-infrared range during non-equilibrium heating of electrons and the lattice [26]. The subsequent decrease in reflection is caused by lattice heating and expansion of the ablation plume.

## 4. Discussion

Irradiation of the surface layer of a metal with an FLP of moderate intensity ~10^13^ W/cm^2^ leads to non-equilibrium heating and subsequent relaxation of electrons and lattice, homogeneous melting of the hot layer within a few picoseconds and expansion of the condensed material. In case of spallation (or photomechanical ablation) [27], cavitation occurs in the molten layer under the action of tensile stresses arising during unloading. The structure of the spallation plume consists of a thin condensed shell filled with a low-density droplet–vapor mixture. Therefore, on a time scale of ~10^−9^ s, so-called Newton’s rings are observed due to the interference between the reflected probe pulse from the semi-transparent spall shell and the crater bottom [28].

As the laser fluence increases, at temperatures close to the critical value (approximately 0.9*T_c_* [29]), a phase explosion occurs in the form of a dense vapor–droplet mixture ejected without the influence of tensile stresses. Large-scale TTM-MD simulations show that the transition from spallation to phase explosion is characterized by a drastic, threshold-like increase in the amount of vapor released into the ablation plume [27,29]. Using pump–probe transient reflectivity microscopy, this transition on picosecond timescales has been experimentally observed to be associated with a sharp drop in reflectivity and with the disappearance of the Newton’s rings, which are otherwise observed on sub-nanosecond timescales in the spallation regime [13,30]. In the intermediate regime, vapor release and stress relaxation act together, causing enhanced fragmentation of matter in the plume.

The mechanism of laser ablation of multilayer films is less studied compared to bulk samples, since it is individual for each structure and depends on many parameters, including layer thickness and sequence, thermophysical and mechanical properties, adhesion strength, etc. Let us consider the obtained experimental data for the investigated multilayer Ni/Al film.

To interpret the experimental results the two-temperature model (TTM) is used [31], which describes the electron $T_{e}(z,t)$ and lattice $T_{i}\left(z,t\right)$ temperature behavior following the ultrafast laser heating:

(2) $$C_{e}\frac{\partial T_{e}}{\partial t}=\frac{\partial }{\partial z}\left(K_{e}\frac{\partial T_{e}}{\partial z}\right)-g\left(T_{e}-T_{i}\right)+S(z,t)=0,$$

(3) $$C_{l}\frac{\partial T_{i}}{\partial t}=g\left(T_{e}-T_{i}\right).$$

The source term $S\left(z,t\right)$ is given by the relation:

(4) $$S(z,t)=0.94\frac{\left(1-R\right)F}{\tau_{p}\delta }exp\left[-\frac{z}{\delta }-2.77{\left(\frac{t}{\tau_{p}}\right)}^{2}\right],$$

where *F* is the incident laser fluence, *z* is the direction normal to the film surface, $\tau_{p}$ = 60 fs is the laser pulse width, δ is the optical penetration depth and *R* = 0.4 is the measured reflectivity of the Ni/Al film sample at the studied laser intensity.

We used a constant electron–phonon coupling parameter g and linear temperature dependences of the electron heat capacity $C_{e}=A_{e}T_{e}$ and thermal conductivity $K_{e}=K_{e0}T_{e}/T_{i}$. Due to the significantly larger laser focal spot size compared to the structure thickness, a one-dimensional (1D) analysis was applied. Laser radiation was absorbed in a skin layer δ=λ/4πk= 13 nm. The parameters used in the calculation and analysis are given in Table 1.

Calculations were performed for the complete 28-layer Ni/Al structure deposited on a glass substrate; on the timescales considered here (≤40 ps) the results were indistinguishable from those obtained for the first two layers only (Ni_1_/Al_1_), since heat did not propagate beyond the first Ni/Al pair within this interval (difference below 0.1%). Zero-heat-flux boundary conditions were applied at the irradiated surface (*z* = 0) and at the bottom of the modeled structure. Equations (2)–(4) were solved numerically using an implicit (backward Euler) finite-volume scheme with a spatial step of 1 nm and a time step of 0.5 fs; inter-nodal conductivities were computed as the harmonic mean of the nodal values, which correctly accounts for the discontinuity of thermal properties at the layer interfaces. At the Ni/Al interface the electron heat flux is continuous and proportional to the electron-temperature jump, −*K_e_*(Ni)·∂*T_e_*/∂*z* = G*_int_*·[*T_e_*(d − 0) − *T_e_*(d + 0)], with G*_int_* = G_0_·*T_e_*/300 K, where G_0_ = 1.71 GW·m^−2^·K^−1^ [35] and *T_e_* is the mean electron temperature of the two sides of the interface (electronic diffuse-mismatch scaling [36]. Because the volumetric lattice heat capacity of aluminum is about 1.7 times lower than that of nickel, the aluminum lattice heats up faster for a comparable electron-to-lattice energy flux. This simple model does not describe the hydrodynamic processes and phase transformations that occur within the fluence range under consideration. Therefore, it cannot be used to quantitatively predict the dynamics of spallation and phase explosion. Nevertheless, it helps us understand the qualitative physical picture of the processes taking place.

The calculations above employ constant values of the electron heat capacity coefficient *A*_e_ and the electron–phonon coupling factor *g*, which are commonly used in TTM-MD simulations of Ni ablation [19,29,37,38]. At the same time, at electron temperatures of 10–25 kK, the constant electron–phonon coupling factor and the linear approximation for *C_e_* are applied beyond the range in which they were originally established. We therefore repeated the calculations using the temperature-dependent electron heat capacity and electron–phonon coupling factor of Ni and Al obtained from first-principles electronic density of states [32]. For Ni, these data give an electron heat capacity 3–7 times smaller than the linear approximation at *T_e_* = 3–25 kK, and a coupling factor decreasing from 1.1 × 10^18^ at 300 K to 0.8 × 10^17^ W·m^–3^·K^–1^ at 25 kK. The peak electron temperature is sensitive to this choice, increasing from 25 to 57 kK at 1 J/cm^2^. At the same time the lattice temperatures, on which our interpretation is based, change more moderately and the qualitative picture used for the interpretation of the experiment—spallation of the molten Ni layer at 0.2 J/cm^2^ and phase explosion of Ni_1_ accompanied by strong overheating of the underlying Al_1_ at 1 J/cm^2^—remains robust with respect to the choice of electronic parameters, although, as noted above, the simple TTM approach cannot be used for quantitative predictions.

**Figure 5:**
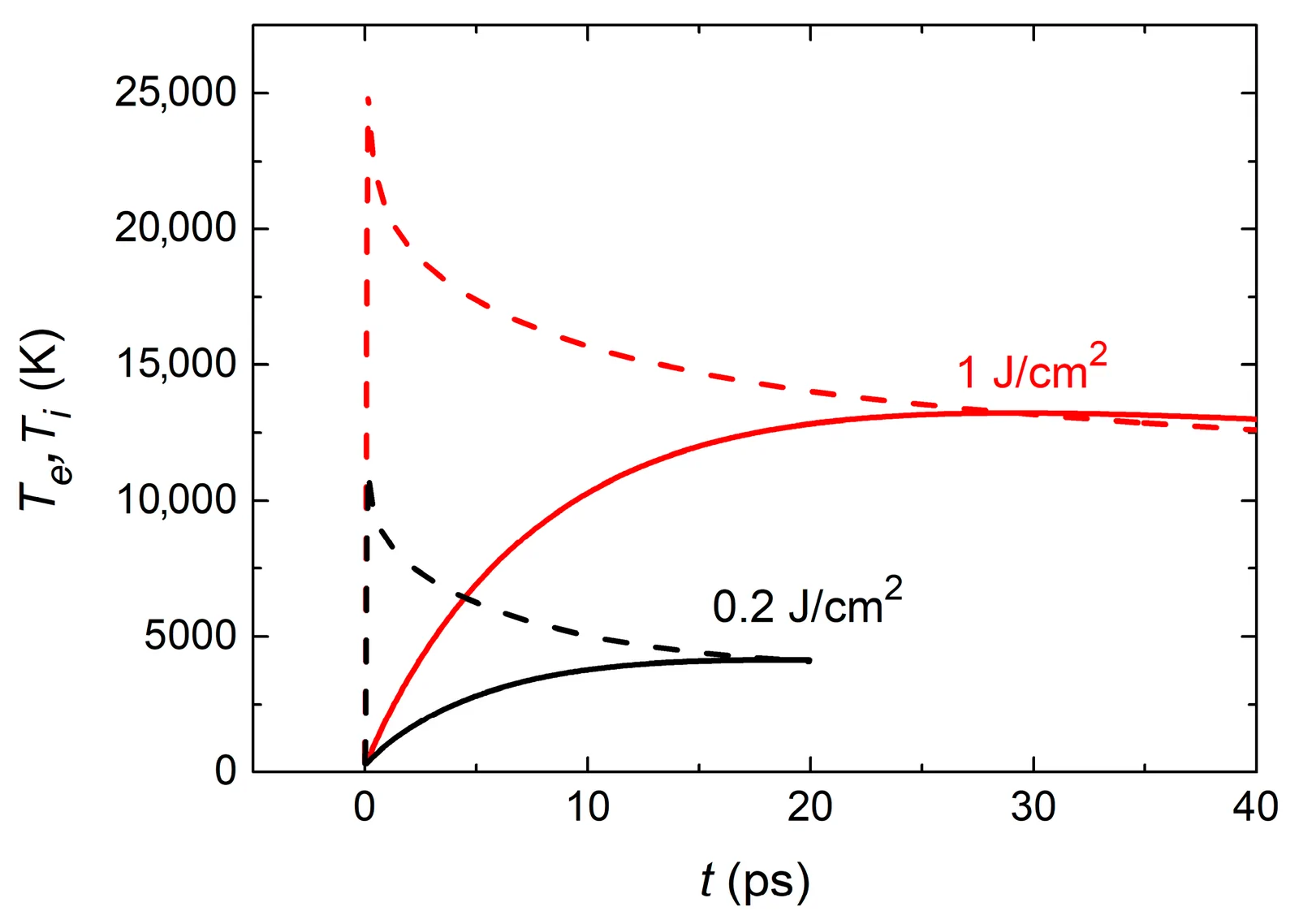
Time dependence of electron (dashed curve) and lattice (solid curve) temperatures predicted by the TTM for the multilayer Ni/Al film irradiated with a 60 fs laser pulse at an incident fluence of 1 J/cm^2^ (red) and 0.2 J/cm^2^ (black).

**Figure 6:**
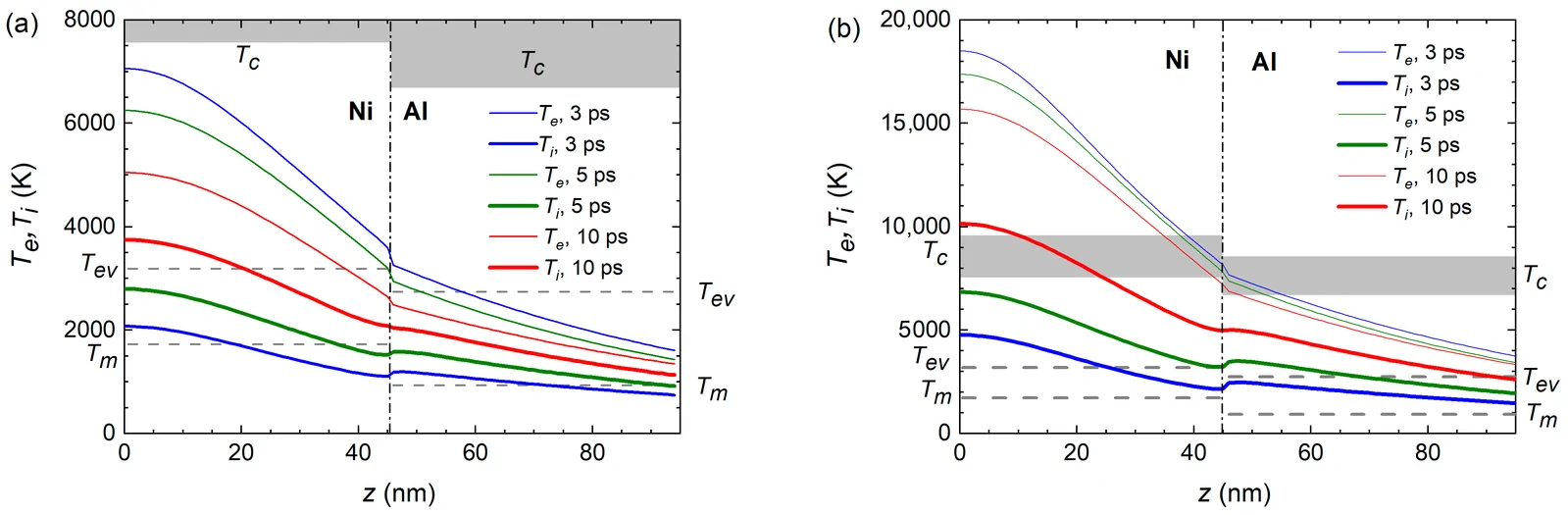
Electron (dashed curves) and lattice (solid curves) temperature distributions in Ni_1_ and Al_1_ layers at different times for incident fluence *F*_0_: (**a**) 0.2 J/cm^2^; (**b**) 1 J/cm^2^. Gray dashed horizontal lines correspond to the melting $T_{m}$ and evaporation $T_{ev}$ temperatures. The range of critical temperature $T_{c}$ is shown in gray.

Figure 5 and Figure 6 show spatiotemporal behavior of temperature, using constant parameters, given in Table 1. Figure 5 illustrates the calculated temporal evolution of the electron and lattice temperatures at the surface of the Ni/Al film for two incident laser fluence values. The maximum electron temperatures at the surface reached 25.1 kK and 11.1 kK for *F*_0_ = 1 J/cm^2^ and *F*_0_ = 0.2 J/cm^2^, respectively. The electron and lattice temperature equilibration times depending on $T_{e}$, scaling with $\tau_{el}\approx C_{e}\left(T_{e}\right)/g$, were 28 ps and 19 ps for the specified fluence values.

At the two-temperature stage, when $t<\tau_{el}$, hot electrons of the Ni_1_ layer, reaching the interface between the layers, transfer energy to the Al electrons and then to the Al lattice [10]. Figure 6 illustrates the calculated distributions of electron and lattice temperatures within the two upper layers of the Ni/Al structure for different fluences.

At an incident fluence of *F*_0_ = 0.2 J/cm^2^, the lattice temperature in both layers is significantly lower than the critical point (see Figure 6a). Therefore, the phase explosion mode does not occur in this case. Meanwhile, the lattice temperature in the nickel layer exceeds the melting point. Thus, spallation occurs in the Ni_1_ layer when a sufficiently high tensile stress causes nucleation in the melt.

At the maximum fluence *F*_0_ = 1 J/cm^2^, the lattice temperature in the Ni_1_ layer reaches $T_{c}$, and conditions for phase explosion can take place. Meanwhile, in the Al_1_ layer, the lattice temperature remains below the critical value but exceeds $T_{ev}$, and the melt should be overheated. Note that the simple TTM overestimates values of the lattice temperature, since it does not take into account the energy costs of melting, vaporization, and hydrodynamic motion of the substance. The enthalpies of melting Δ*H*_m_ for Ni and Al are 17.6 kJ/mol and 10.8 kJ/mol, respectively. The scale of the overestimated values Δ*T_i_* = Δ*H*_m_/*C_l_* is 675 K for Ni and 443 K for Al. Above *T_m_*, the model overestimates the lattice temperature at 1 J/cm^2^ for Ni and Al by approximately 5% and 6% of the maximum *T_i_* values, respectively, while at 0.2 J/cm^2^ the overestimation reaches 16% and 19%. Under these conditions, the lattice temperatures of Ni and Al are expected to remain above *T_m_*.

**Figure 7:**
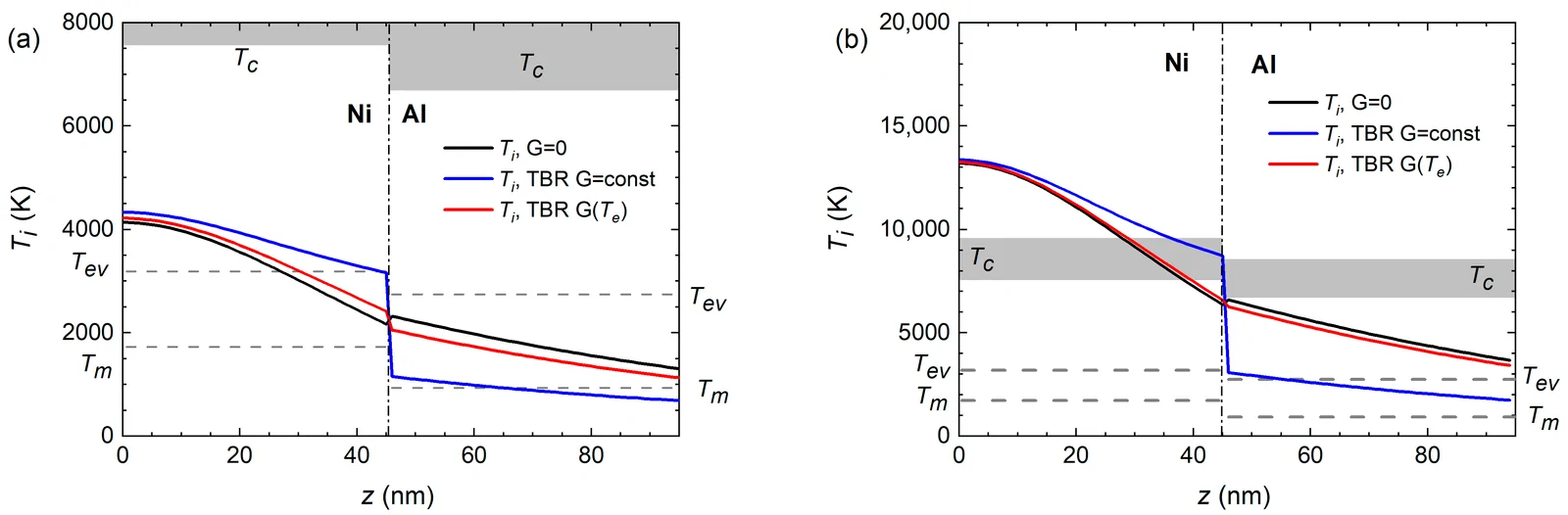
Lattice temperature distributions in Ni_1_ and Al_1_ layers after electron-lattice relaxation for incident fluence *F*_0_: (**a**) 0.2 J/cm^2^, *t* = 19 ps; (**b**) 1 J/cm^2^, *t* = 28 ps; without TBR (G = 0, black curve) and taking into account TBR: G = 1.71 GW·m^–2^·K^–1^ (blue curve); G = 1.71·*T_e_*/300 GW·m^–2^·K^–1^ (red curve). The range of critical temperature $T_{c}$ is shown in gray.

The issue of thermal contact at the Ni/Al interface, specifically the continuity of electron temperature and heat flux, requires more thorough consideration. The only direct measurement of the thermal boundary conductance of Al/Ni interfaces that we are aware of [35] gives G ≈ 1.7–2.6 GW·m^–2^·K^–1^ (*R_int_* = 1/G ≈ (4–6) × 10^–10^ m^2^·K·W^–1^), i.e., comparable to the intrinsic thermal resistance of the 46 nm Ni layer (R ≈ 5 × 10^−10^ m^2^·K·W^–1^, Bi ≈ 0.8–1.2). For other metal–metal interfaces, G can reach 4–20 GW·m^−2^·K^−1^ [36]. To assess the sensitivity of our results to this assumption, we performed the calculations with G = 0 GW·m^−2^·K^−1^ (an ideal thermal contact) and a finite interface conductance G = 1.7 GW·m^−2^·K^−1^, both as a constant value and scaled with electron temperature according to the electronic diffuse-mismatch model [11] (Figure 7).

For G = 1.71·*T_e_*/300 GW·m^–2^·K^–1^ (this is the case for electron and lattice temperature distributions in Figure 6), the peak electron temperature and the maximum lattice temperature of the Ni_1_ layer change by less than 6% compared to ideal case; the lattice temperature of Al_1_ layer at *F*_0_ = 1 J/cm^2^ decreases somewhat but remains above the evaporation temperature of Al, while at *F*_0_ = 0.2 J/cm^2^ it remains above the melting temperature. The qualitative conclusions of this study are therefore unaffected by the finite interfacial thermal resistance.

The lower acoustic impedance of aluminum, compared to nickel, leads to interference of rarefaction waves, moving inside the Ni_1_ layer towards each other from the free surface and the boundary between the layers [10]. With an impedance ratio of $\mu =Z_{Al}/Z_{Ni}\approx$ 0.34 [36], the reflection coefficient of an acoustic wave at the interface is equal to $R=\left(1-\mu \right)/(\left.1+\mu \right)\approx$ 0.49. As a result of interference, the spallation threshold $F_{a1}\;»$ 0.07 J/cm^2^ is lower compared to a bulk nickel 0.3 J/cm^2^ [25].

The ablation dynamics typical for spallation [15] is shown in Figure 4, curve *1*. For a fluence of *F* = 0.2 J/cm^2^ the recorded phase shift of approximately 0.1 rad during the first picoseconds is caused by a change in the optical constants upon heating and the onset of motion of the surface [24]. The subsequent phase shift after 20 ps is mainly determined by the movement of the spall layer at a velocity of 0.32 km/s. The expansion rate was determined from the slope of the $\Delta \varphi_{t}\left(t\right)$ dependence, taking into account relation (1). The uncertainty in velocity estimation was determined by linear fit function and did not exceed 10%.

The similar expansion dynamics were detected in the range $F_{a1}<F<F_{a2}$, in which spallation mode for the Ni/Al film was realized. In the specified range a partial or complete removal of the Ni_1_ layer took place (Figure 3, crater profile). Near the threshold $F_{a1}$ spallation occurs within the Ni_1_ layer at a minimum depth of 28 nm from the surface. As the fluence increases, the spall depth grows. In the vicinity $F_{a2}$ crater depth reaches the boundary of Ni_1_/Al_1_ layers. The estimations show that the Al_1_ layer should be in a liquid state in this case. However, the ejection of molten aluminum may be suppressed by the pressure created by the expanding plume [9].

In the case of $F>F_{a2}$, a significant, jet-like increase in the ejection of matter was observed (Figure 3). At the maximum energy density of $F_{0}=$ 1 J/cm^2^, the recorded large phase shift of 1.35 rad in 6 ps and a sharp drop in the reflectivity of the probe pulse (Figure 4, curves *2*, *4*) should be caused by the rapid expansion of a highly scattering vapor or vapor–droplet mixture. A similar behavior of the optical signal—reflectance oscillations due to interference within the spalled layer in the spallation regime, followed by their disappearance upon the transition to phase explosion—was recently reported for FeNi targets, where combined pump–probe measurements and atomistic simulations directly linked these optical signatures to the underlying ablation dynamics [39]. Comparable transient reflectivity drops accompanying melting, spallation, and phase explosion have also been observed on pure nickel films using time-resolved reflectivity microscopy [30], supporting our interpretation of the observed optical response. A related transition in optical response was also reported for a thin chromium film, where time-resolved topography revealed a shift from a smooth, spallation-related surface relief to a rougher, phase-explosion-related morphology accompanied by a stronger drop in reflectivity [40]. In the Au/Ti bilayer system studied by pump–probe imaging combined with TTM-MD simulations [13], the gold overlayer was found to delay and dampen the optical response associated with the vapor expansion of the underlying titanium layer, analogous to the way the upper Ni_1_ layer in our sample modulates the optical signal produced by the ablation of the underlying Al_1_ layer. This consistency across chemically and structurally different systems supports our interpretation that the observed abrupt phase and reflectivity changes in the Ni/Al film suggest the same generic transition from a stress-driven (spallation) to a vapor-dominated (phase explosion) ablation regime.

According to TTM calculations, this is associated with the transition from spallation to the phase explosion mode upon reaching nickel critical temperature. The subsequent slower expansion of the material after approximately 40 ps is likely caused by the ejection of overheated molten aluminum. Due to the large velocity gradients and rapid drop in vapor density, the nickel vapor pressure in the plume becomes insufficient to suppress the expansion of the underlying Al_1_ layer and part of the overheated molten aluminum layer is removed, forming a 19 nm deep recess (Figure 3).

A rough estimate using the relation (1) shows that, if the entire measured phase shift is attributed to surface motion, the apparent initial expansion rate over the first few picoseconds could reach ~9 km/s. This value should, however, be regarded only as an upper bound, since the phase shift contains additional contributions from fluence-dependent changes in the optical constants and scattering within the ablation plume, especially at high fluence where the reflected probe signal strongly decreases. Subsequent expansion of the matter after 40 ps occurs at a velocity of approximately 1 km/s. It should be emphasized that a rigorous solution to the problem of material expansion under the specified conditions requires numerical hydrodynamic modeling that takes into account the spatially non-uniform density profile [41], which was not the objective of this study.

Thus, the experimental results for the multilayer Ni/Al thin-film indicate a spallation regime for the removal of the upper Ni_1_ layer due to stress localization in the melt. At the two-temperature stage electrons of nickel effectively transfer the absorbed FLP energy from the skin layer to the underlying aluminum, which has a lower melting point. The accumulation of energy in the Al_1_ layer seems to leads to an explosive release of overheated molten aluminum after exceeding the corresponding threshold value. Removing a thin top layer from an underlying one with a lower melting point requires precise control of the processing parameters.

## 5. Conclusions

The ultrafast dynamics of the layer-by-layer ablation of a Ni/Al thin film irradiated with a 60 fs, 800 nm femtosecond laser pulse have been studied using spatially and temporally resolved spectral interferometry. This work is focused on the initial stage of ablation in a time interval from 0 to 120 ps. The top 46 nm thick Ni_1_ layer was partially or fully removed in spallation mode due to localized stress in the melt. The corresponding spall layer velocity reached 0.3–0.4 km/s. Near the spallation threshold of $F_{a1}=$ 0.07 J/cm^2^ the minimum crater depth was 28 nm. The complete separation of the top layer occurred at a fluence range of 0.4–0.6 J/cm^2^. The intense jet-shaped ejection of matter was observed above the second ablation threshold $F_{a2}=$ 0.6 J/cm^2^, which suggests the complete removal of the upper nickel layer in the phase explosion mode and the subsequent explosive expansion of the underlying overheated molten aluminum. The obtained results may be of interest for studying the physics of heat transfer during non-equilibrium stage in multilayered nanomaterials upon irradiation with ultrashort laser pulses, investigating the ablation mechanisms, developing simulations and optimizing laser processing.

## Figures and Tables

**Table 1:** Material constants. The values for electron–phonon coupling constant *g* are taken from [32]; thermal conductivities $K_{e0}$ and specific heat constants $A_{e}$ are from [19]; lattice heat capacities $C_{l}\;$ are from [33], melting $T_{m}$ and evaporation $T_{ev}$ temperatures are from [25]; critical temperatures for Ni and Al are from [6,29,34].

	*g* (10^17^ Wm^−3^K^−1^)	*K_e_*_0_ (Wm^−1^K^−1^)	*A_e_* (Jm^−3^K^−2^)	*C_l_* (10^6^ Jm^−3^K^−1^)	*T_m_* (K)	*T_ev_* (K)	*T_c_* (K)
Ni	3.6	91	1065	4.1	1728	3186	7585–9576
Al	3.1	237	135	2.42	933	2743	6700–8550

## Data Availability

The original contributions presented in this study are included in the article. Further inquiries can be directed to the corresponding author.

## Abbreviations

The following abbreviations are used in this manuscript:

FLP	Femtosecond laser pulse
TTM	Two-temperature model
